# B-Cell Activation Gene Signature in Blood and Liver of Hepatitis B e Antigen–Positive Patients With Immune Active Chronic Hepatitis B

**DOI:** 10.1093/infdis/jiae280

**Published:** 2024-06-07

**Authors:** Zgjim Osmani, Boris J B Beudeker, Zwier M A Groothuismink, Robert J de Knegt, Raymond T Chung, Jeroen Aerssens, Jacques Bollekens, Harry L A Janssen, Adam J Gehring, Georg M Lauer, Alex K Shalek, Harmen J G van de Werken, Andre Boonstra

**Affiliations:** Department of Gastroenterology and Hepatology, Erasmus University Medical Center, Rotterdam, The Netherlands; Department of Gastroenterology and Hepatology, Erasmus University Medical Center, Rotterdam, The Netherlands; Department of Gastroenterology and Hepatology, Erasmus University Medical Center, Rotterdam, The Netherlands; Department of Gastroenterology and Hepatology, Erasmus University Medical Center, Rotterdam, The Netherlands; Liver Center, Division of Gastroenterology and Liver Center, Massachusetts General Hospital and Harvard Medical School, Boston, Massachusetts; Clinical Translational Science Infectious Diseases, Janssen Research and Development, Beerse, Belgium; Clinical Translational Science Infectious Diseases, Janssen Research and Development, Beerse, Belgium; Department of Gastroenterology and Hepatology, Erasmus University Medical Center, Rotterdam, The Netherlands; Toronto Centre for Liver Disease, Toronto General Hospital, University of Toronto; Toronto Centre for Liver Disease, Toronto General Hospital Research Institute, University Health Network; Department of Immunology, University of Toronto, Ontario, Canada; The Ragon Institute of Massachusetts General Hospital, Massachusetts Institute of Technology and Harvard University; The Ragon Institute of Massachusetts General Hospital, Massachusetts Institute of Technology and Harvard University; Institute for Medical Engineering and Science, Department of Chemistry, and Koch Institute for Integrative Cancer Research, Massachusetts Institute of Technology; Broad Institute of Massachusetts Institute of Technology and Harvard, Cambridge, Massachusetts; Department of Immunology, Erasmus University Medical Center, Rotterdam, The Netherlands; Department of Gastroenterology and Hepatology, Erasmus University Medical Center, Rotterdam, The Netherlands

**Keywords:** B cells, chronic hepatitis B, liver fine-needle aspirates, single-cell RNA sequencing, activation gene signature

## Abstract

**Background:**

Studies on chronic hepatitis B virus (HBV) infection have shown immune dysfunction involving multiple cell types, including T cells. B cells have been evaluated more recently, but in contrast to T cells, more pronounced activation of circulating B cells has been reported. To gain more insight into the activation status of B cells, we investigated gene profiles of B cells in the blood and liver of patients with chronic HBV.

**Methods:**

RNA-sequencing and flow cytometric analysis was performed on peripheral blood B cells of patients with immune active chronic HBV, comparing them with samples from healthy controls. In addition, gene expression profiles of B cells in the liver were analyzed by bulk and single-cell RNA-seq.

**Results:**

Our data show a distinctive B-cell activation gene signature in the blood of patients with immune active chronic HBV, characterized by a significant upregulation of immune-related genes. This peripheral activation profile was also observed in B cells from the liver by single-cell RNA-seq, with naive and memory B-cell subsets being the primary carriers of the signature.

**Conclusions:**

Our findings suggest that B-cell gene profiles reflect responsiveness to HBV infection; these findings are relevant for clinical studies evaluating immunomodulatory treatment strategies for HBV.

## Teaser

The role of B cells in controlling chronic hepatitis B virus (HBV) infection is still not well established. We show that, in contrast to other cell types, a distinctive B-cell activation gene signature is found in patients with immune active chronic HBV.

## Graphical abstract

**Graphical abstract:**
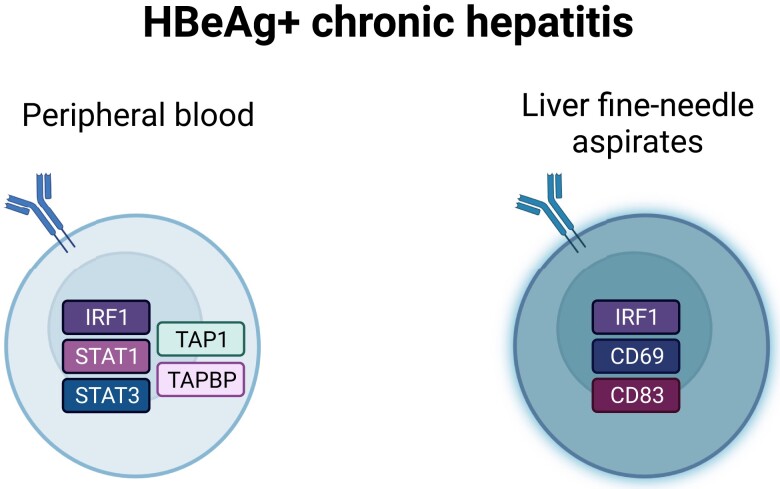

Persistent infections with the hepatitis B virus (HBV) continue to pose a significant worldwide health challenge, leading to the development of liver cirrhosis, end-stage liver disease, and hepatocellular carcinoma [1]. Immunological studies on chronic HBV infection have shown a plethora of immune defects, such as hampered dendritic cell function and impaired functional T-cell responses [2]. Recently, we compared liver samples from nucleoside analogue–treated and untreated patients with chronic HBV and identified significant differences in the single-cell transcriptome of myeloid cells, including numerous immune-related genes, whereas B cells exhibited few significant changes [3]. This indicated that despite ongoing HBV replication and changes in the liver immune environment, B-cell gene profiles remain relatively unchanged. However, in contrast to T cells, other studies have reported on altered or activated B-cell responses in chronic HBV infections (reviewed in [4]). For instance, elevated protein levels of activation markers CD69 and CD71 were reported on peripheral blood B cells of patients with chronic HBV when compared to healthy controls [5, 6]. Also, microarray analysis of whole blood indicated that innate interferon genes and B-cell response signatures were highly active during the hepatitis B e antigen–positive (HBeAg^+^) immune tolerant and immune active phases of chronic HBV, respectively [7]. However, since HBV replication takes place exclusively in the liver, evaluation of the intrahepatic compartment is essential to determine the status of B cells during chronic HBV infections. Previously, we observed low levels of B-cell infiltration in clinical phases with low serum alanine aminotransferase (ALT) and viral load levels, but extensive infiltration of B cells was observed in liver biopsies of HBeAg^+^ patients with immune active chronic HBV, predominantly around the portal and periportal areas [8, 9]. The observed infiltration correlated significantly with serum ALT levels, reflecting liver inflammation, but not with HBV DNA levels [9]. However, little information is available on the activation status of B cells during the immune active clinical phase with high serum ALT and viral load levels. Considering that novel antiviral agents alone are insufficient for the eradication of HBV, and the potential necessity for immunomodulatory treatments to restore effective immunologic control [10], it is important to better understand the parameters determining B-cell responsiveness in the liver to unveil their potential role and utilization in novel immunomodulatory therapeutic approaches. Therefore, in this study, we aimed to compare the gene expression profiles of blood and liver B cells in patients with HBeAg^+^ chronic hepatitis and compare these to healthy controls. Given the formation of B-cell infiltrates, our objective was to unravel whether discernible B-cell activation patterns in HBeAg^+^ immune active chronic hepatitis can be identified.

## METHODS

Detailed methodology for patient inclusion, sample processing, and analysis is provided in the Supplementary Materials and Methods (Supplementary Table 1) [3, 9, 11, 12].

## RESULTS

### B-Cell Activation Gene Signature in Peripheral Blood B Cells of HBeAg^+^ Patients With Chronic Hepatitis

**Figure 1.**
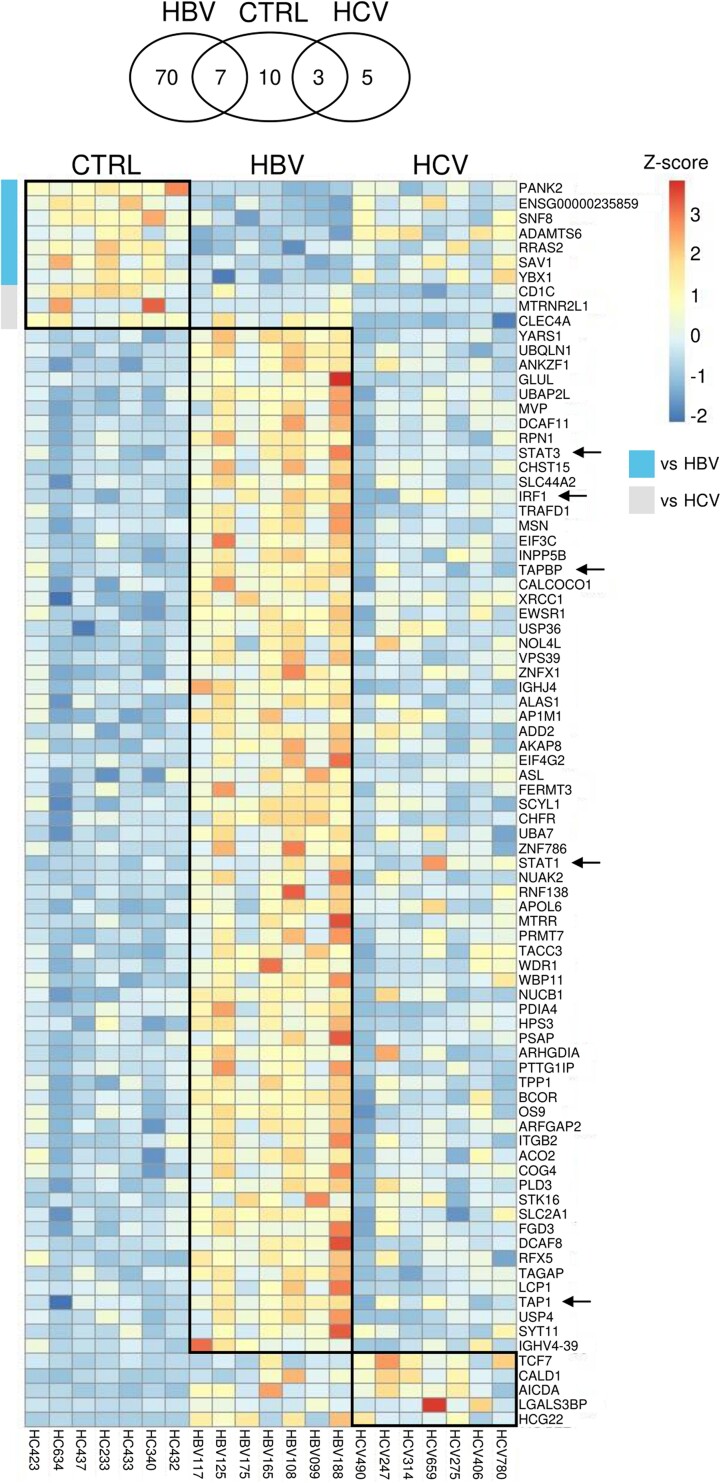
Venn diagram and heatmap showing the number of significantly differentially expressed genes (DEGs) observed in RNA-sequencing data of peripheral blood B cells comparing healthy controls (CTRL) versus patients with chronic hepatitis B virus (HBV) or hepatitis C virus (HCV) (Wald test, adjusted *P* < .05). The color scale represents the Z-scores of DEGs. Arrows point toward immune-related genes (*IRF1*, *STAT1*, *STAT3*) and genes involved in antigen presentation (*TAP1*, *TAPBP*).

**Figure 2.**
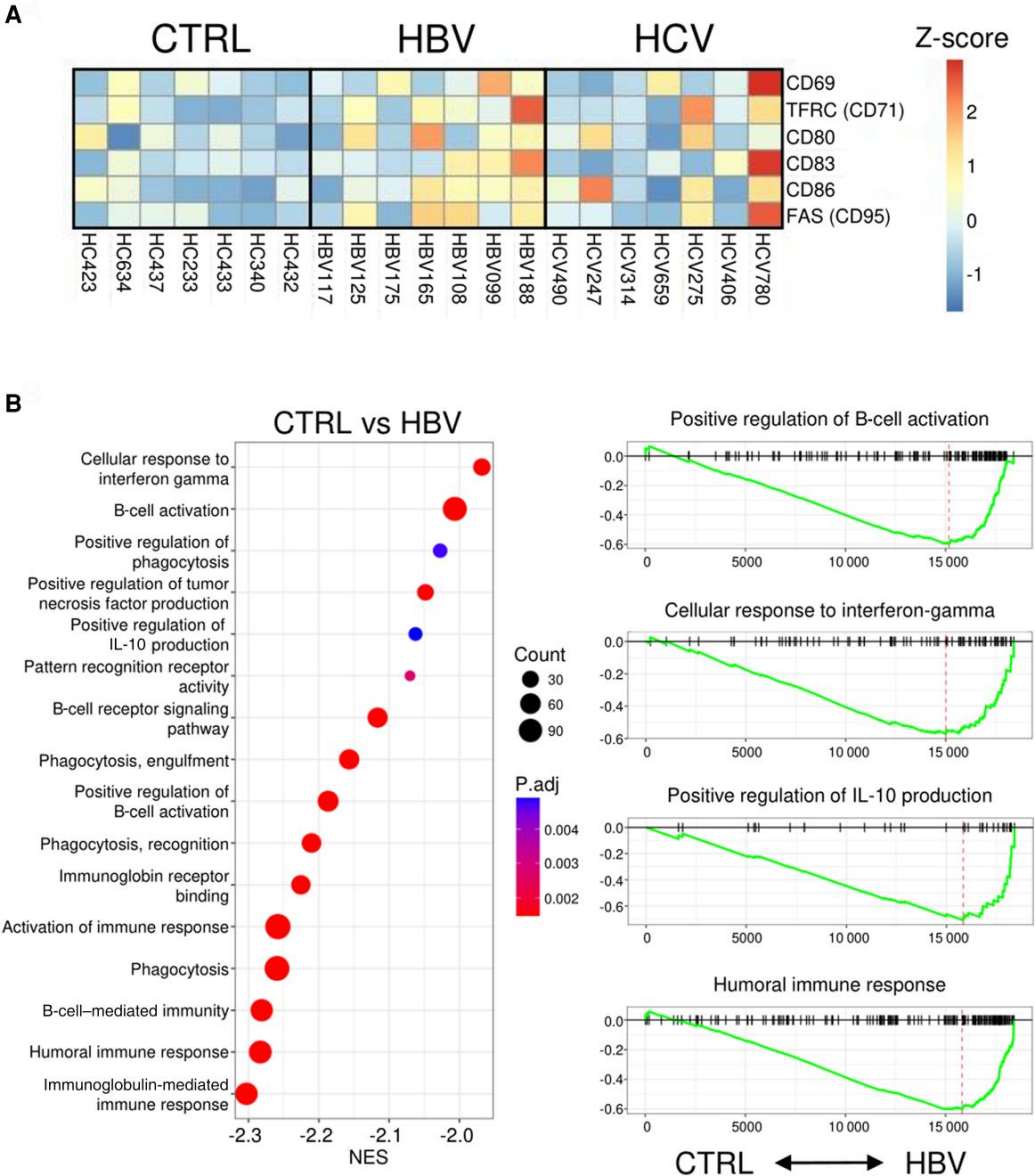
*A*, Heatmap displaying gene expression levels of B-cell activation markers in RNA-sequencing data of peripheral blood B cells of healthy controls (CTRL) and patients with chronic hepatitis B virus (HBV) or hepatitis C virus (HCV). The color scale represents the Z-scores. *B*, Gene set enrichment analysis (GSEA) plots show gene sets and corresponding normalized enrichment scores (NES) significantly enriched in the CTRL versus HBV peripheral blood B cells. Gene sets with negative NES are increased in the HBV group. The color and size of dots represent the adjusted *P* values and gene count, respectively. On the right side of the figure, GSEA plots are shown for a selection of gene sets. The vertical lines in the running enrichment score show where the gene set members appear in the ranked list of genes. The dotted line represents the maximum enrichment score.

Previously, we showed an enrichment of B-cell gene signatures (eg, *CD79A*, *IGHD*, *TCL1A*, *VPREB3*) in the peripheral blood of HBeAg^+^ patients with immune active chronic HBV [7]. None of the detected gene expression signatures were indicative of B-cell activation. However, this study relied on microarray gene expression analysis of RNA extracted from whole blood, which has limited sensitivity and resolution. Therefore, we employed a more sensitive approach using RNA sequencing (RNA-seq) on purified peripheral blood CD19^+^ B cells from healthy controls and HBeAg^+^ patients with immune active chronic HBV. In addition, we included a control group of patients with chronic viremic HCV with elevated ALT levels to differentiate between systemic effects of liver inflammation on peripheral blood B cells and changes specific to chronic HBV infection (Table 1). A total of 70 genes were statistically significantly higher expressed by B cells of patients with chronic HBV as compared to the healthy control group (Figure 1, Supplementary Table 2), including higher expression of immune-related genes (*IRF1*, *STAT1*, *STAT3*) and genes involved in antigen presentation (*TAP1*, *TAPBP*). In contrast to chronic HBV, only 5 genes were expressed at significantly higher levels by B cells of chronic HCV patients as compared to the healthy control group, which did not overlap with the transcriptome profile of B cells from patients with chronic HBV. In addition, only 7 and 3 genes were shown to be significantly higher expressed by B cells of the healthy control group as compared to patients with chronic HBV or HCV, respectively. Moreover, relatively higher expression levels of B-cell activation genes *TFCR* (CD71), *CD80*, *CD83*, *CD86*, and *FAS* (CD95) were observed in 4 of 7 patients with chronic HBV as compared to healthy controls (Figure 2*A*, HBV099, 108, 165, 188). Differences in the transcriptome of B cells in patients with chronic HBV coincided with a significant enrichment of gene sets involved in, among others, the cellular response to interferon-γ, humoral immune responses, and the positive regulation of B-cell activation and interleukin 10 production (Figure 2*B*). In summary, our findings show that transcriptomic profiles in purified B cells were distinctive to chronic HBV infection, with higher expression levels of B-cell activation genes, in line with previous studies [5, 6]. The induction of a type I interferon signature in B cells may indicate B-cell activation and the initiation of an antiviral response; however, neither HBV nor HCV significantly induced the expression of interferon-stimulated genes (ISGs) in peripheral blood B cells (data not shown).

**Table 1.** Patient Characteristics of RNA-Sequencing Data From Peripheral Blood Purified CD19^+^ B Cells

Characteristic	Controls	HBV	HCV
Samples, No.	7	7	7
Age, y, median (IQR)	34 (32–37)	38 (31–41)	47 (40–54)
Male sex	4 (57%)	4 (57%)	4 (57%)
Race			
White	2 (28.6%)	1 (14.3%)	2 (28.6%)
Asian	3 (42.8%)	5 (71.4%)	3 (42.8%)
African	2 (28.6%)	0 (0%)	1 (14.3%)
Other	0 (0%)	1 (14.3%)	1 (14.3%)
ALT, U/L, median (IQR)	NA	44 (36–163)	47 (29–58)
Viremia, log_10_ IU/mL, median (IQR)	NA	8.7 (8.0–9.0)	6.3 (6.2–6.9)
HBeAg positive	NA	7 (100%)	NA
Anti-HBe positive	NA	0 (0%)	NA
HBV genotype	NA	…	NA
A	…	2 (28.5%)	…
B	…	1 (14.3%)	…
C	…	3 (42.9%)	…
ND	…	1 (14.3%)	…
HCV genotype	NA	NA	…
HCV-1	…	…	1 (14.3%)
HCV-1a	…	…	1 (14.3%)
HCV-1b	…	…	4 (57%)
HCV-4	…	…	1 (14.3%)
Fibrosis			
F0/F1	…	5 (71.4%)	6 (85.7%)
F2	…	2 (28.5%)	1 (14.3%)

Data are presented as No. (%) unless otherwise indicated.

Abbreviations: ALT, alanine aminotransferase; HBeAg, hepatitis B e antigen; HBV, hepatitis B virus; HCV, hepatitis C virus; IQR, interquartile range; NA, not applicable.

### Significant Enrichment of Atypical Memory B Cells in Blood of Patients With Chronic HBV

**Figure 3.**
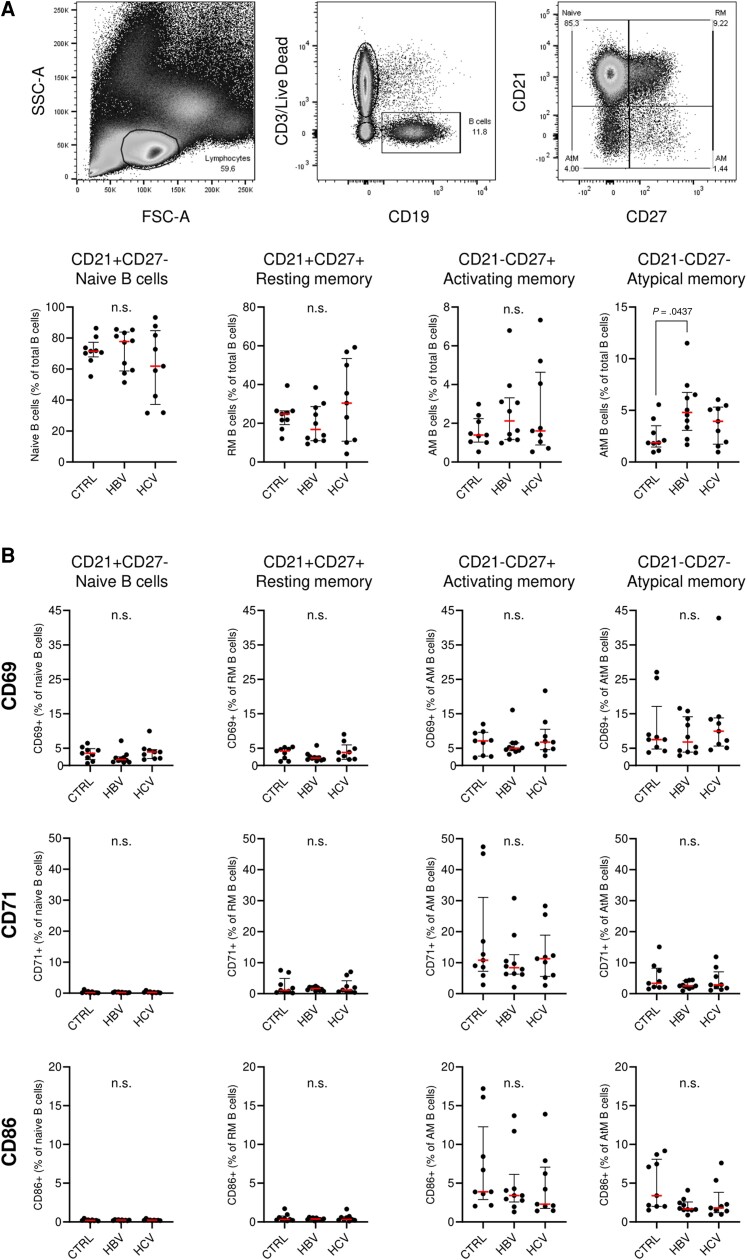
*A*, Gating strategy and flow cytometric analysis comparing peripheral blood B-cell subsets of healthy controls (CTRL) versus patients with hepatitis B virus (HBV) or hepatitis C virus (HCV), including naive (CD21^+^CD27^−^), resting memory (RM; CD21^+^CD27^+^), activating memory (AM; CD21^−^CD27^+^), and atypical memory (AtM; CD21^–^CD27^−^) B cells. *B*, Flow cytometric data showing the percentage of positive cells for activation markers CD69, CD71, and CD86 in peripheral blood B-cell subsets of CTRL versus HBV and HCV. Dunn multiple comparisons test was applied for statistical analyses (*P* < .05); n.s., not significant.

To understand whether the increased expression of B-cell activation-related genes in patients with chronic HBV could be attributed to frequency differences in subsets of circulating B cells and to translate these findings to the protein level, we conducted flow cytometric analysis on peripheral blood B-cell subsets in HBeAg^+^ patients with immune active chronic HBV and patients with chronic viremic HCV, comparing results with healthy controls (Supplementary Table 3). For the analysis, chronic HBV and HCV patients with high viral loads and ALT levels were selected. Interestingly, we observed no significant differences in the frequency of naive, resting memory, and activating memory (AM) B-cell subsets (Figure 3*A*, Supplementary Table 4), except for a significant 2.5-fold enrichment of atypical memory (AtM) B cells in the blood of patients with chronic HBV as compared to the healthy control group (4.8% vs 1.9%, respectively; Dunn multiple comparisons *P* = .0437). The overall expression of CD83 on B-cell subsets was typically low, usually <1%. AM and AtM B cells exhibited the highest expression of the early B-cell activation marker CD69, while activation markers CD71 and CD86 were more highly expressed by AM B cells alone (Figure 3*B*). Nevertheless, we did not observe a distinct trend indicating higher activation marker expression by B-cell subsets in the blood of patients with chronic HBV or HCV as compared to the healthy control group (Supplementary Table 4, Supplementary Figure 1).

### RNA-seq Analysis of Liver Tissues From HBeAg^+^ Patients With Immune Active Chronic HBV Shows Increased Expression of *CD79B*, *CD38*, and Immunoglobulin Genes as Compared to Healthy Controls

**Figure 4.**
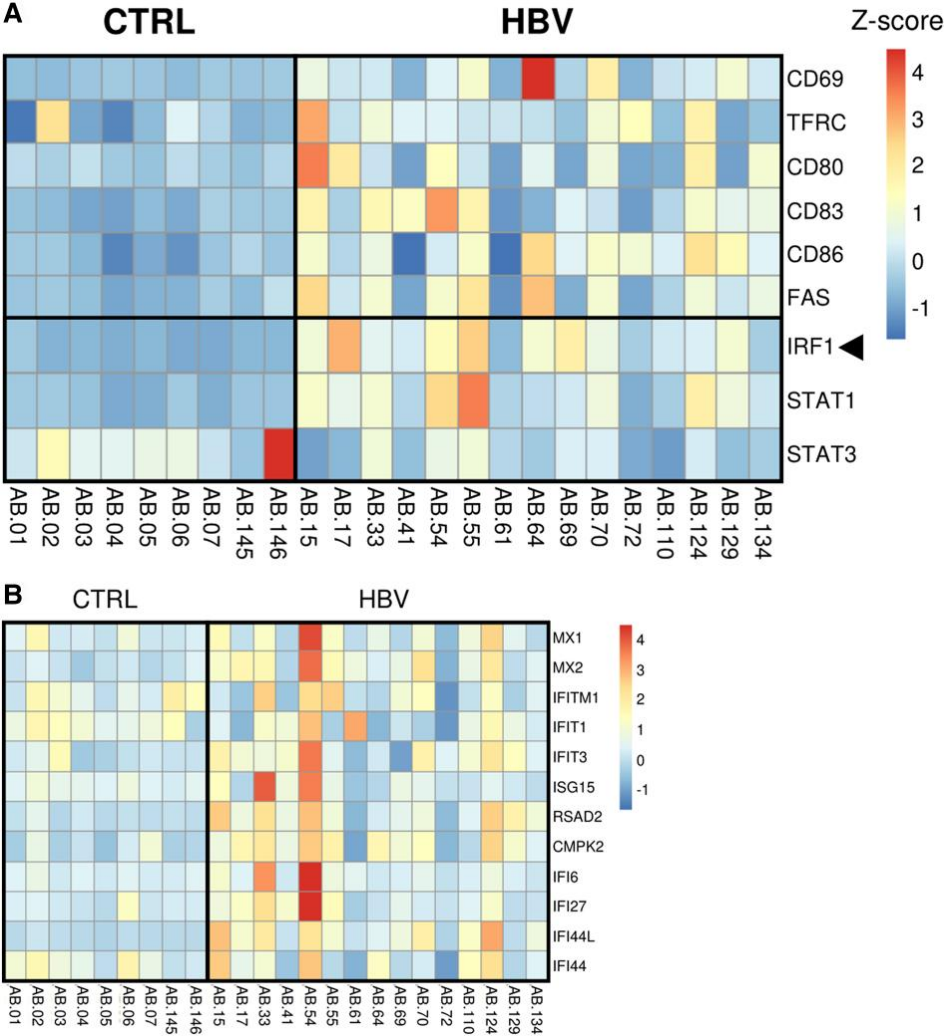
*A*, Heatmap displaying gene expression levels of B-cell activation markers in RNA-sequencing (RNA-seq) data of liver biopsies from healthy controls (CTRL) and patients with chronic hepatitis B virus (HBV). *B*, Relative gene expression levels of interferon-stimulated genes observed in RNA-seq data of liver biopsies from CTRL and HBV. The color scale represents the Z-scores.

In addition to the systemic response, investigating local immune responses to HBV in the liver is crucial, as viral replication occurs in infected hepatocytes and B-cell infiltrates are detected. As the phenotype and function of intrahepatic B cells might differ from their peripheral counterparts, we reanalyzed RNA-seq data of liver biopsies to evaluate if B-cell activation gene signatures could be identified in the liver of HBeAg^+^ patients with immune active chronic HBV compared to healthy controls (Supplementary Table 5) [9]. We identified a total of 1680 differentially expressed genes, with 1111 genes that were significantly more highly expressed in patients with chronic HBV (Supplementary Table 6). Genes that, according to our findings and previous studies, are associated with B-cell activation such as *CD69*, *TFRC* (CD71), *CD80*, *CD83*, *CD86*, *FAS* (CD95), *IRF1*, and *STAT1* were expressed at relatively low levels in liver tissues of the healthy control group (Figure 4*A*). Interestingly, we observed higher expression levels of these B-cell activation genes in 7 of 15 liver tissues of patients with chronic HBV (AB.15, 33, 54, 55, 64, 70, 124). Yet no significant differences were observed for these genes between the chronic HBV and the healthy control group, except for *IRF1* (adjusted *P* = .0051; Figure 4*A*). In addition, we identified that 33 of 1680 genes (2%) were B-cell related and showed significant differential expression between liver tissues of patients with chronic HBV and healthy controls (Supplementary Table 7). For instance, *CD79B*, *CD38*, *IGHM*, *IGHG2/G4*, and *IGHA1/A2* expression levels were significantly higher in chronic HBV liver tissues than in the healthy control group. The higher gene expression levels of B-cell activation genes in the liver of patients with chronic HBV may, at least in part, be attributed to a higher frequency of infiltrating B cells, as reported previously [9]. To note, there was a modest, albeit not significant, induction of ISGs in liver tissues of patients with chronic HBV as compared to the healthy control group (Figure 4*B*).

### Single-Cell Transcriptomic Profiles of Total B Cells in the Liver of HBeAg^+^ Immune Active Chronic HBV Patients Is in Line With the Observation of Active B-Cell Gene Profiles in Blood

**Figure 5.**
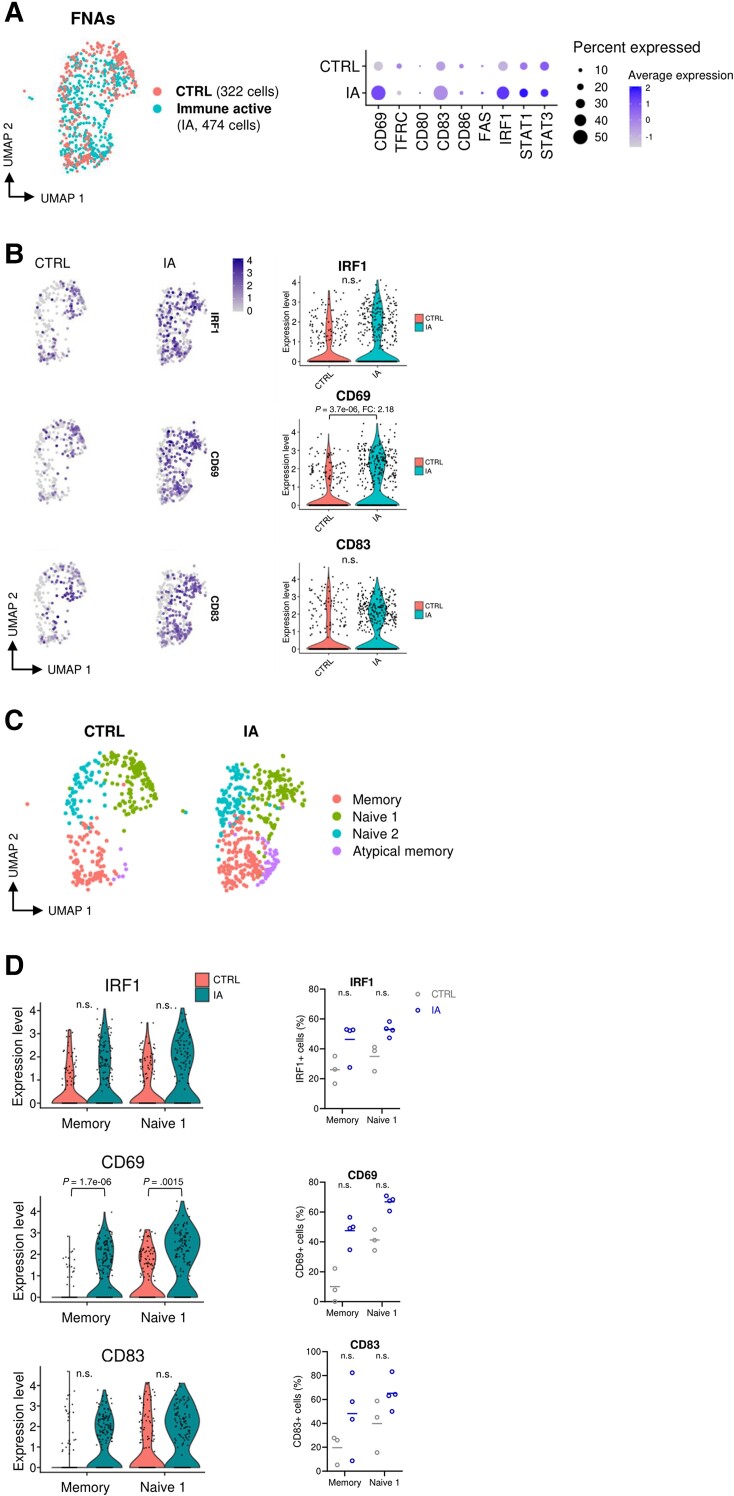
*A*, The dot plot of total intrahepatic B cells captured from the liver of healthy controls (CTRL; n = 322) and patients with immune active (IA) chronic hepatitis B (n = 474) are shown in red and blue, respectively. Each dot represents a single cell. On the right, a feature dot plot shows the log average gene expression levels of B-cell activation markers and immune-related genes (*IRF1*, *STAT1*, *STAT3*) in total intrahepatic B cells of CTRL and patients with IA chronic hepatitis. The size represents the percentage of cells expressing the gene of interest and the color scale represents the log average expression levels. *B*, Feature plots showing *IRF1*, *CD69*, and *CD83* log gene expression levels. Each dot represents a single cell, and the relative log gene expression levels are represented by a color scale and violin plots on the right side of the figure. *C*, Dot plot of intrahepatic B-cell clusters identified in the liver of CTRL and patients with IA chronic hepatitis, including a naive 1, naive 2, memory, and atypical memory B-cell cluster. *D*, Violin plots showing the relative log gene expression levels of *IRF1*, *CD69*, and *CD83* in the memory and naive 1 B-cell cluster of CTRL and patients with IA chronic hepatitis are shown in red and blue, respectively. On the right, the percentage of cells expressing *IRF1*, *CD69*, and *CD83* in the memory and naive 1 B-cell cluster of CTRL and patients with IA chronic hepatitis are shown in gray and blue, respectively. Significant *P* values were obtained with the Wilcoxon rank-sum test (Bonferroni adjusted *P* < .05). Abbreviations: CTRL, healthy controls; FC, fold change; FNA, fine-needle aspirate; IA, immune active; n.s., not significant; UMAP, uniform manifold approximation and projection.

Liver tissues from HBeAg^+^ patients with immune active chronic HBV revealed a notable increase in the expression of B-cell activation-associated markers. To overcome the limitations of bulk RNA-seq, we profiled intrahepatic B cells using single-cell RNA-seq (scRNA-seq) on liver fine-needle aspirates (FNAs) obtained from HBeAg^+^ patients with immune active chronic HBV and healthy controls (Table 2). Despite the limitations of FNAs yielding a relatively low absolute number of intrahepatic immune cells, and an even scarcer number of B cells, a total of 474 and 322 B cells were captured from the aspirates of the chronic HBV and healthy control groups, respectively (Figure 5*A*). Differential gene expression analysis comparing total B cells from liver showed that 11 genes were significantly more highly expressed in patients with immune active chronic HBV as compared to the healthy control group, including *CD69*, *KLF6*, and *IGHG2-4* (Supplementary Table 8). Furthermore, 10 genes in total B cells from the healthy control group exhibited significantly higher expression levels, including *CD96*, *USP53*, and *TSPAN13*, among others (Supplementary Table 8). Interestingly, increased gene expression levels of *IRF1*, *CD69*, *CD83*, and, to a lesser extent, *STAT1/3*, were observed in total B cells from patients with immune active chronic HBV compared with the healthy control group (Figure 5*A* and 5*B*). In contrast to the bulk liver RNA-seq data, we observed homogeneous gene expression levels of B-cell activation genes in all 4 patients with immune active chronic hepatitis B. This further supports our previous observations that distinct alterations can be found in transcriptomic profiles of intrahepatic B cells in chronic HBV infection, pointing toward higher expression levels of B-cell activation-associated genes.

**Table 2.** Patient Characteristics of Single-Cell RNA-Sequencing Data From Fine-Needle Aspirate B Cells

Characteristic	Control	Immune Active
Samples, No.	3	4
Age, y	22, 61, 64	28, 33, 45, 50
Male sex	2 (66%)	1 (25%)
Race		
White	3 (100%)	…
Asian	…	4 (100%)
African	…	…
Other	…	…
ALT, U/L	…	85, 136, 198, 256
HBV DNA, log_10_ IU/mL	…	7.7, 7.9, 8.0, 8.2
HBeAg positive	…	4 (100%)
Anti-HBe positive	…	0 (0%)
F0/F1 fibrosis	…	4 (100%)

Data are presented as No. (%) unless otherwise indicated.

Abbreviations: ALT, alanine aminotransferase; HBeAg, hepatitis B e antigen.

### Both Naive and Memory B Cells in the Liver of HBeAg^+^ Patients With Immune Active Chronic HBV Reveal an Active B-Cell Gene Profile

As previously reported, B cells obtained from the liver can be divided into 4 clusters: naive 1, naive 2, memory, and an AtM B-cell cluster (Figure 5*C*) [11]. We show that in patients with immune active chronic HBV, both the naive 1 and memory B-cell clusters display higher gene expression levels of *IRF1* (fold change [FC]: 2.1 and 1.7, respectively) and *CD83* (FC: 1.3 and 1.3) and significantly higher *CD69* expression (FC: 2.5 and 3.2) when compared to the healthy control group, as indicated by the respective fold changes (Figure 5*D*, Supplementary Table 8). In addition, the proportions of naive 1 and memory B cells expressing *IRF1*, *CD69*, and *CD83* were relatively higher in patients with immune active chronic HBV as compared to the healthy control group (Figure 5*D*). The analysis of AtM B cells was precluded by the limited absolute number of cells. Again, there was no induction of ISGs in intrahepatic B cells of patients with chronic HBV (data not shown).

## DISCUSSION

HBeAg^+^ chronic hepatitis is associated with intrahepatic B-cell infiltrates; however, their activation profiles in the liver are not well characterized. We aimed to explore the activation gene profile of B cells in the blood and liver of HBeAg^+^ patients with immune active chronic HBV to increase our understanding of their role in immunity against HBV. Our study provides a comprehensive analysis of B cells and B-cell activation, utilizing different RNA-seq datasets and thereby offering an unbiased perspective. We show that (1) a more pronounced B-cell activation gene signature is induced in the blood of patients with chronic HBV as compared to healthy controls, and (2) scRNA-seq of the liver supports the activation profile as observed by RNA-seq in blood and identifies naive and memory B-cell clusters in the liver as the most activated subsets. In line with previous studies [5–7], these findings suggest that B-cell gene profiles in blood and liver are indicative of B-cell activation and their responsiveness to HBV during HBeAg^+^ chronic hepatitis. This introduces the possibility that unlike T cells, B cells maintain responsiveness both systemically and in the liver, where viral replication occurs and B-cell infiltrates can be observed. However, by flow cytometry we were unable to show that both HBV and HCV induced protein expression of activation markers in B-cell subsets as compared to healthy controls, which is in part consistent with a study conducted by Ni et al [13]. Furthermore, we show that HBV does not significantly induce ISGs in B cells from blood and liver of patients with HBeAg^+^ immune active chronic HBV (data not shown). This observation aligns with the previously reported absence of a prominent interferon response in peripheral blood natural killer cells [14].

The finding obtained by evaluating blood and liver samples that, in contrast to T cells, B cells indeed show an activation gene signature during HBeAg^+^ chronic hepatitis, is an important one. It places the B-cell compartment aside from all other immune cell types in chronic HBV. It is important to stress that our study assessed the total B-cell population and not HBV-specific B cells, since especially for hepatitis B surface antigen (HBsAg)–specific B cells there is no evidence for a higher activation status as observed by us and others [15–17]. Furthermore, our current study was unable to determine the trigger for the activated state. In peripheral blood of HBeAg^+^ patients with chronic hepatitis, we did not observe a significant correlation between HBsAg or ALT levels and immune-related gene expression profiles in B cells (data not shown). We demonstrated before that B-cell infiltration in the liver of HBeAg^+^ patients with chronic hepatitis correlated significantly with ALT levels, and not HBV viral load [9]. Previously, we observed correlations between ALT levels and intrahepatic gene expression levels of *IRF1*, *STAT1*, *IGHG1*, and *IGHG4* [9]. It is possible that B-cell activation may be the result of events taking place in the inflamed liver that are not directly related to the amount of virus present. One possibility is that the HBsAg or other soluble factors associated with inflammation, such as cytokines, may be involved in modulating the activity of B cells. In this regard, it is important to mention that recently our group reported that gene profiles of memory B cells in the blood and liver of nucleoside analogue–treated patients with chronic HBV showed signs of increased activation associated with high serum HBsAg levels, as shown by scRNA-seq [18]. Although more studies need to be conducted to determine the underlying mechanisms, these observations further suggest that B cells may be activated by HBV not dependent directly on viral replication, and may point toward a role for HBsAg levels in inducing immune cell activation. It is important to acknowledge that our study design did not include the measurement of HBV RNA or HBV core-related antigen levels in the blood of HBeAg^+^ patients with chronic hepatitis, which could also potentially modulate the activity of B cells.

The role of B cells in modulating or controlling chronic HBV infection is still not well established. It is known for many years that treatment with anti-CD20 monoclonal antibody rituximab poses a significant increased risk of HBV reactivation [19], illustrating the importance of B cells in the continuous control of HBV, even during a stable chronic HBV infection. Complex interactions of B cells with other immune cells likely determines the outcome of immune response to the virus in chronically infected patients. The best illustration of this comes from liver biopsies that demonstrate follicle-like structures and immune cell aggregates consisting of multiple immune cell types [9]. In blood of patients with chronic HBV, follicular helper T cells (Tfh) are enriched and are thought to be involved in HBV-specific immune responses by promoting B-cell differentiation, thereby regulating B-cell–mediated humoral immune responses against HBV [20]. This involvement may lead to increased B-cell activation with higher expression levels of activation markers such as CD69, CD71, CD83, and CD86 on B cells of patients with chronic HBV [4]. In addition, natural killer cells have also been reported to modulate B-cell responses by enhancing B-cell activation and antibody production through cytokine secretion, thereby promoting antiviral immunity [21]. Also, inhibition of B-cell responses through direct killing of activated B cells or indirectly by suppressing Tfh cell responses has been described, limiting excessive immune activation and potential immunopathology [21]. Further studies are needed to understand the exact mechanisms and interactions involved, as well as to explore their effects on functional B-cell responses such as antibody production, antigen presentation, or cytokine production. These insights may guide future therapeutic strategies aimed at enhancing immune responses against HBV.

## Supplementary Data

Supplementary materials are available at *The Journal of Infectious Diseases* online (http://jid.oxfordjournals.org/). Supplementary materials consist of data provided by the author that are published to benefit the reader. The posted materials are not copyedited. The contents of all supplementary data are the sole responsibility of the authors. Questions or messages regarding errors should be addressed to the author.

## Supplementary Material

jiae280_Supplementary_Data

## Notes

***Author contributions.*** Z. O. and A. B. contributed to the design of the research. Z. O. and Z. M. A. G. carried out the experiments and procedures. A. B. and H. J. G. v. d. W. were involved in planning and supervised the work. B. J. B. B., R. T. C., R. J. d. K., H. L. A. J., J. A., and J. B. were involved in clinical study design and setup and/or enrollment of the patients. R. T. C., R. J. d. K., H. L. A. J., J. A., J. B., A. J. G., G. M. L., A. K. S., and A. B. worked on conceptual development. Z. O. was responsible for conducting the bioinformatic analysis and wrote the manuscript. All authors discussed the results, provided critical feedback, revised the manuscript, and contributed to the final manuscript.

***Financial support*.** This study was sponsored by the Foundation for Liver and Gastrointestinal Research (SLO) and in part funded by Janssen Pharmaceuticals. R. T. C. was supported by the Massachusetts General Hospital Research Scholars Program. A. J. G. received support from the Toronto Centre for Liver Disease and the Canada Foundation for Innovation. A. K. S. receives funding from the National Institutes of Health, Break Through Cancer, the Pew-Stewart Scholars Program, Foundation MIT, Wellcome Leap, the Bill & Melinda Gates Foundation, the Chan Zuckerberg Initiative, and the Moore Foundation.
