# Supplementary material for: B-Cell Activation Gene Signature in Blood and Liver of Hepatitis B e Antigen–Positive Patients With Immune Active Chronic Hepatitis B
Source: J Infect Dis. 2024 Jun 7;230(6):e1263–73. doi: 10.1093/infdis/jiae280 (PMC11646581; doi:10.1093/infdis/jiae280)
Supplement: jiae280_Supplementary_Data [file jiae280_supplementary_data.zip › 20240518_Supplementary_Material_and_Methods.pdf]

Supplementary Material and Methods

B cell activation gene signature in blood and liver of HBeAg+ immune active chronic hepatitis B patients

Zgjim Osmani[1], Boris J.B. Beudeker[1], Zwier M.A. Groothuismink[1], Robert J. de Knegt[1], Raymond T. Chung[2], Jeroen Aerssens[3], Jacques Bollekens[3], Harry L.A. Janssen[1,4], Adam J. Gehring[5,6], Georg M. Lauer[7], Alex K. Shalek[7,8,9], Harmen J.G. van de Werken[10]*, Andre Boonstra[1]*

*shared last authorship

[1] Department of Gastroenterology and Hepatology, Erasmus University Medical Center, Rotterdam, the Netherlands.

[2] Liver Center, Division of Gastroenterology and Liver Center, Massachusetts General Hospital and Harvard Medical School, Boston, Massachusetts, USA.

[3] Clinical Translational Science Infectious Diseases, Janssen Research and Development, Beerse, Belgium.

[4] Toronto General Hospital, University of Toronto, Ontario, Canada.

[5] Toronto Centre for Liver Disease, Toronto General Hospital Research Institute, University Health Network, Toronto, Ontario, Canada.

[6] Department of Immunology, University of Toronto, Toronto, Ontario, Canada.

[7] The Ragon Institute of Massachusetts General Hospital, Massachusetts Institute of Technology and Harvard University, Cambridge, Massachusetts, USA.

[8] Institute for Medical Engineering and Science (IMES), Department of Chemistry, and Koch Institute for Integrative Cancer Research, Massachusetts Institute of Technology, Cambridge, Massachusetts, USA.

[9] Broad Institute of MIT and Harvard, Cambridge, Massachusetts, USA.

[10] Department of Immunology, Erasmus University Medical Center, Rotterdam, the Netherlands.

Corresponding author: Andre Boonstra, p.a.boonstra@erasmusmc.nl, Wytemaweg 80, 3015 CN Rotterdam, PO Box 2040, 3000 CA Rotterdam.

Alternate corresponding author: Zgjim Osmani, z.osmani@erasmusmc.nl, Wytemaweg 80, 3015 CN Rotterdam, PO Box 2040, 3000 CA Rotterdam.

Patient cohorts

Liver fine-needle aspirate (FNA) samples were obtained from three different sites, including the Erasmus MC (Rotterdam, The Netherlands), Toronto General Hospital (Toronto, Canada) and Massachusetts General Hospital (Boston, USA). All other patient samples were collected from patients recruited at the outpatient clinic of the Erasmus MC Rotterdam. HBeAg+ immune active chronic HBV patients with high viral loads and ALT levels were selected in this study, in accordance with the EASL Clinical Practice Guidelines[1]. Patients did not have other liver diseases, were not pregnant, and were not on antiviral therapy. Liver fibrosis was determined by histology or transient elastography. Patients had no or minimal liver fibrosis at the time of inclusion (maximum of ≤F2, <7.0 kPa). Peripheral blood mononuclear cells (PBMCs) were isolated from patients and healthy control individuals from the Erasmus MC by Ficoll-Paque density gradient centrifugation, and samples were cryopreserved at -150 ^{o}C. The collection of liver FNAs and subsequent sample preparation was performed as described previously[2]. Liver biopsies from chronic HBV patients were collected and archived formalin-fixed paraffin-embedded (FFPE) blocks were stored as part of routine diagnostics at the Erasmus MC. Patients received neither antiviral treatment prior to biopsy, nor had any co-existing primary liver disease, nor were they co-infected with HCV, HEV, HDV or HIV. Liver biopsies of healthy individuals were collected to determine their eligibility as altruistic liver donors. For RNA sequencing and flow cytometric analysis of peripheral blood B cells, all infected cohorts were matched by age, sex, and ethnicity to healthy control individuals. This study was conducted according to the guidelines of the Declaration of Helsinki and the principles of Good Clinical Practice. The study was approved by institutional review boards at all sites. All participants provided written informed consent.

Fluorescence-activated cell sorting and RNA isolation of peripheral blood CD19+ B cells

Frozen PBMCs were thawed and stained for CD3-PE (Clone: UCHT1, BioLegend, cat.: 300408), CD19-eF450 (Clone: HIB19, eBioscience, cat.: 48-0199-42), and Fixable Aqua Dead Cell Stain (Invitrogen, cat.: L34957). CD19+ B cells were isolated from each sample by fluorescence-activated cell sorting using the BD FACSAria II cell sorter. The purities of all samples included in the analysis were higher than 95%. Sorted B cells were kept in TriPure™ Isolation Reagent (Roche, cat.: 11667165001) at -20°C, and RNA was isolated using a PicoPure RNA isolation kit (Arcturus, cat.: KIT0204) according to the manufacturer's protocol. The concentration, quality and integrity of the RNA samples were analyzed on a Bioanalyzer 2100 (Agilent Technologies); all samples had a RIN value of ≥7. Isolated RNA samples were stored at -80°C until sequencing.

RNA sequencing of peripheral blood purified CD19+ B cells

Library preparation and sequencing were performed on the total RNA of sorted CD19+ peripheral blood B cells in one batch by GenomeScan (Leiden, The Netherlands) using the low input RNA sequencing protocol (NEBNext Low Input RNA Library Prep Kit for Illumina). Samples were sequenced paired-end 150 bp with the Illumina NovaSeq 6000 (30 million reads/sample). Prior to sequencing, library preparation QC was performed to check for ribosomal, globin, and mitochondrial content. All samples had 0% globin, <6% mitochondrial and <0.3% ribosomal content. Sequence reads were trimmed to remove possible adapter sequences and filtered on low-quality bases using fastp (v0.23.2). The reads were filtered based on the base quality score to remove noise introduced by sequencing errors. Reads of which more than 40% of the bases had an average phred score below Q15 or which had more than 5 unknown bases were removed. If the resulting reads were shorter than 15 bp, they were removed altogether (both pairs in paired-end reads).

RNA-seq differential gene expression analysis of peripheral blood CD19+ B cells

For each sample, trimmed reads were mapped to the human reference genome GRCh38.p13 using a short read aligner STAR2 (v2.7.10) with default settings. Based on the mapped locations in the alignment file, the frequency of reads mapped on a transcript was determined with HTSeq (v2.0.2). Unique reads that fall within exon regions were counted, and count files generated with HTSeq were used as input for downstream analysis. Prior to differential expression analysis, we performed pre-filtering of genes to keep only the genes with a minimum count of 10 for a minimal number of 3 samples. A total of 18,418 out of 40,693 detected genes (45.3%) passed the filtering criteria. Differential expression analysis was performed with DESeq2 (v1.32.0) using the Wald test. Benjamini-Hochberg adjusted p-values were considered significant if <0.05. To enable comparison of gene expression across all samples outside of the context of differential expression analysis, DESeq2 normalized counts were calculated and scaled to obtain Z-scores. Gene expression analyses between the healthy control (CTRL) and chronic HBV group were further assessed for overrepresenting gene sets related to biological states or processes. Results were analyzed as a ranked list of genes, sorted by fold changes in decreasing order. A normalized enrichment score (NES) was calculated for each gene set, reflecting the degree to which it is overrepresented at the top or bottom of the ranked list. P-values were calculated based on permutations using the R-package clusterProfiler (v3.18) and were corrected with the Benjamini-Hochberg FDR correction method.

Flow cytometric immunophenotyping of peripheral blood B cells

Frozen PBMCs were thawed, and washed with RPMI 1640 supplemented with 10% fetal calf serum (FCS; Lonza, Walkersville, MD, USA). For flow cytometry, at least $3x10^6$ viable PBMC were incubated for 30 minutes at 4°C in the dark with a mixture of anti-CD19, CD3, CD27, CD21, CD38, CD69, CD71, CD83, and CD86 antibodies including a fixable viability stain and Fc block (**Suppl. Table 1**). The staining buffer contained PBS+1% FCS and 10% v/v Super Bright Complete Staining Buffer (eBioscience, SB-4401-75). Cells were washed twice with PBS+1% FCS prior to acquisition. Data was acquired with the BD FACS Symphony flow cytometer, and analyzed using FlowJo version 10.9.0 (Tree Star Inc.).

RNA isolation and sequencing of liver tissue biopsies

Previously, our group performed RNA sequencing of FFPE core needle liver biopsies[3]. Total RNA isolation and library preparation were performed as described previously[3]. Libraries for each sample were sequenced at EA Genomics (Q2 solutions, USA) on the Illumina HiSeq platform (100 bp paired-end reads, 50 million reads per sample).

Differential gene expression analysis of liver tissue biopsies

Raw paired-end reads were aligned to the human reference genome hg38 using STAR (v.2.7.3a), as described previously[3]. Picard tools were employed to detect at least 10 million reads in protein coding mRNA regions for downstream analysis. Prior to differential gene expression analysis, we performed pre-filtering of genes to keep only the genes with a minimum count of 10 for a minimal number of 3 samples. Differentially expressed genes (DEGs) were calculated with DESeq2 (v1.32.0) using the Wald test (≥1.5 FC). Benjamini-Hochberg adjusted p-values <0.05 were considered significant. To enable comparison of gene expression across all samples outside of the context of differential expression analysis, DESeq2 normalized counts were calculated and scaled to obtain Z-scores.

Single-cell RNA sequencing analysis of B cells from liver fine-needle aspirates

Liver fine-needle aspirates (FNAs) were obtained from four HBeAg+ immune active chronic HBV patients and three healthy control (CTRL) individuals. All samples were sequenced using the Seq-Well S^3 platform. Libraries were pre-processed using the DropSeq pipeline with the tools v2.3.0 as previously described[4]. Prior to single-cell RNA sequencing (scRNA-seq) analysis, low-quality cells or empty droplets were filtered by removing cells with <300 features and >20% of reads mapped to the mitochondrial genome. Cell

doublets or multiplets were removed by excluding cells with >6000 features. B cell clusters were annotated manually, based on well-known lineage-specific markers retrieved from differentially expressed genes (DEGs) using the FindAllMarkers function (min.pct=0.1, logfc.threshold=0.6, only.pos=TRUE, p.adj<0.05), as described previously[5]. The Wilcoxon rank-sum test was applied to assess significant DEGs comparing the single-cell transcriptome of individual B cell clusters and total B cells between chronic immune active (IA) HBV patients (n=474 cells) vs. CTRL (n=322 cells). To assign significant DEGs, genes expressed by at least 10% of cells in one of both groups were included and a cut-off value of 1.5 for absolute fold change was applied (Bonferroni p.adj <0.05).

Statistical analyses

R (version 4.2.2) and GraphPad Prism (version 8) were used for statistical analyses. Continuous variables were expressed as median with interquartile range (IQR). The Wilcoxon rank-sum or Dunn's multiple comparisons test was applied when appropriate for statistical analyses of cell frequencies and flow cytometric data. Differential expression analysis of bulk RNA sequencing (RNA-seq) data was performed using the Wald test, and the Wilcoxon rank-sum test was used for scRNA-seq data. Results were considered statistically significant if the (adjusted) p-values were <0.05.

Data availability statement

Bulk RNA-seq data of peripheral blood purified CD19+ B cells is publicly available and was deposited in Gene Expression Omnibus (GEO, https://www.ncbi.nlm.nih.gov/geo/) with accession number: GSE250153. RNA-seq data of liver biopsies is publicly available (GSE230397). Single-cell sequencing data of intrahepatic B cells from FNAs of chronic immune active HBV and CTRL will be made available upon reasonable request.

References

1. European Association for the Study of the Liver. Electronic address eee, European Association for the Study of the L. EASL 2017 Clinical Practice Guidelines on the management of hepatitis B virus infection. J Hepatol **2017**; 67:370-98.
2. Genshaft AS, Subudhi S, Keo A, et al. Single-cell RNA sequencing of liver fine-needle aspirates captures immune diversity in the blood and liver in chronic hepatitis B patients. Hepatology **2023**; 78:1525-41.
3. Montanari NR, Ramírez R, Aggarwal A, et al. Multi-parametric analysis of human livers reveals variation in intrahepatic inflammation across phases of chronic hepatitis B infection. J Hepatol **2022**; 77:332-43.
4. Hughes TK, Wadsworth MH, 2nd, Gierahn TM, et al. Second-Strand Synthesis-Based Massively Parallel scRNA-Seq Reveals Cellular States and Molecular Features of Human Inflammatory Skin Pathologies. Immunity **2020**; 53:878-94 e7.
5. Osmani Z, Arreola Villanueva M, Joseph-Chazan J, et al. Intrahepatic plasma cells, but not atypical memory B cells, associate with clinical phases of chronic hepatitis B, **manuscript accepted in the European Journal of Immunology (2024)**.
