# Supplementary material for: B-Cell Activation Gene Signature in Blood and Liver of Hepatitis B e Antigen–Positive Patients With Immune Active Chronic Hepatitis B
Source: J Infect Dis. 2024 Jun 7;230(6):e1263–73. doi: 10.1093/infdis/jiae280 (PMC11646581; doi:10.1093/infdis/jiae280)
Supplement: jiae280_Supplementary_Data [file jiae280_supplementary_data.zip › 20240520_Suppl_Figures_and_tables.pdf]

Supplemental figures and tables

B cell activation gene signature in blood and liver of HBeAg+ immune active chronic hepatitis B patients

Zgjim Osmani[1], Boris J.B. Beudeker[1], Zwier M.A. Groothuismink[1], Robert J. de Knegt[1], Raymond T. Chung[2], Jeroen Aerssens[3], Jacques Bollekens[3], Harry L.A. Janssen[1,4], Adam J. Gehring[5,6], Georg M. Lauer[7], Alex K. Shalek[7,8,9], Harmen J.G. van de Werken[10]*, Andre Boonstra[1]*

**shared last authorship*

[1] Department of Gastroenterology and Hepatology, Erasmus University Medical Center, Rotterdam, the Netherlands.
[2] Liver Center, Division of Gastroenterology and Liver Center, Massachusetts General Hospital and Harvard Medical School, Boston, Massachusetts, USA.
[3] Clinical Translational Science Infectious Diseases, Janssen Research and Development, Beerse, Belgium.
[4] Toronto General Hospital, University of Toronto, Ontario, Canada.
[5] Toronto Centre for Liver Disease, Toronto General Hospital Research Institute, University Health Network, Toronto, Ontario, Canada.
[6] Department of Immunology, University of Toronto, Toronto, Ontario, Canada.
[7] The Ragon Institute of Massachusetts General Hospital, Massachusetts Institute of Technology and Harvard University, Cambridge, Massachusetts, USA.
[8] Institute for Medical Engineering and Science (IMES), Department of Chemistry, and Koch Institute for Integrative Cancer Research, Massachusetts Institute of Technology, Cambridge, Massachusetts, USA.
[9] Broad Institute of MIT and Harvard, Cambridge, Massachusetts, USA.
[10] Department of Immunology, Erasmus University Medical Center, Rotterdam, the Netherlands.

Table of content

Supplemental figure..……………………………………… p.1
Supplemental tables………………………………………. p.2-9

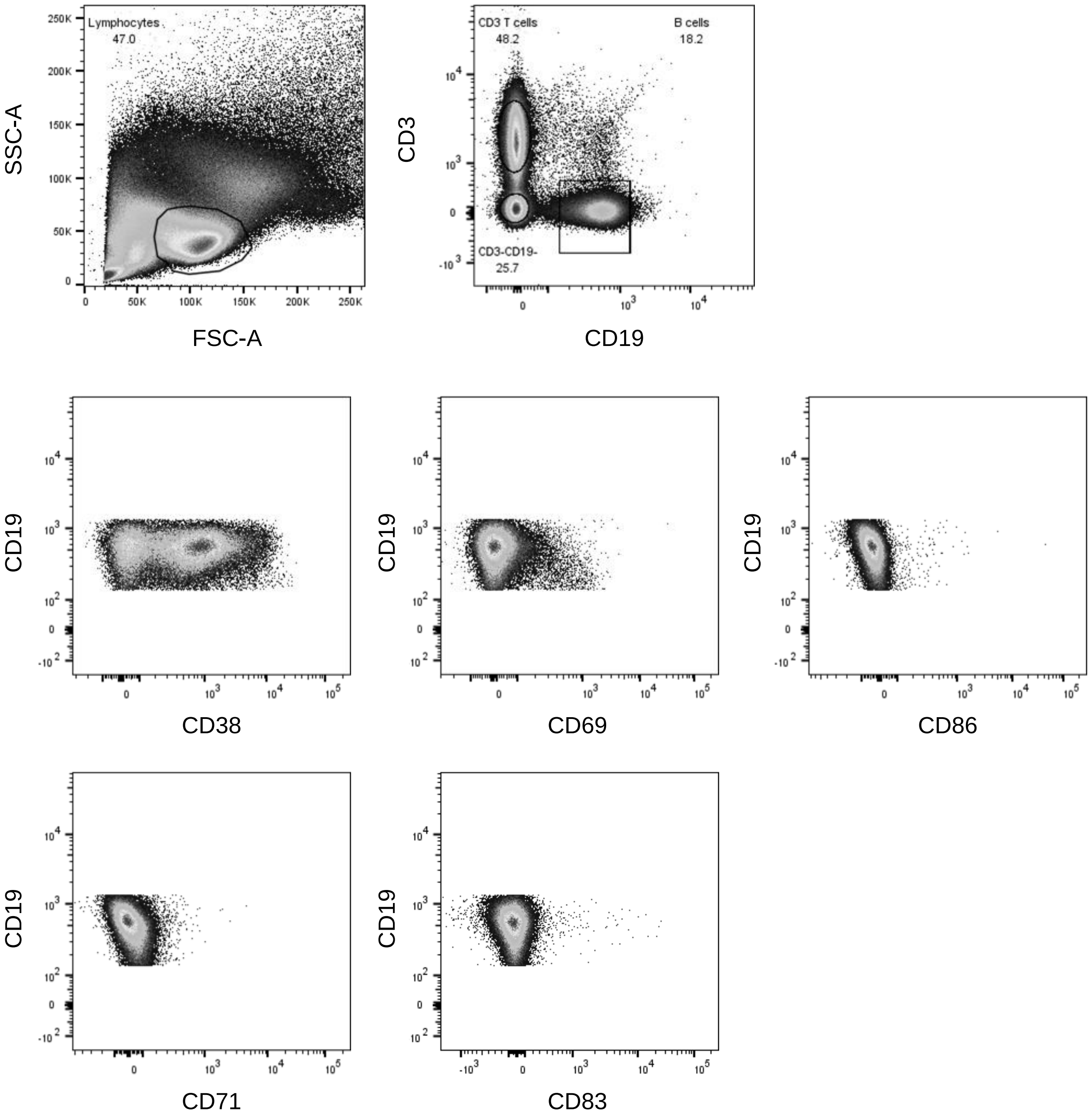

Suppl. Figure 1. Representative FACS plots showing the flow cytometric analysis of activation markers in peripheral blood CD19+ B cells from frozen PBMCs samples.

Suppl. Table 1. Antibody cocktail used for flow cytometric analysis of peripheral blood B cells.

Name	Supplier	Cat no.	Clone no.
Fc block	BD Pharmingen	564219	n/a
Fixable Viability Stain 575V	BD Horizon	565694	n/a
CD27 FITC	BD Biosciences	340424	L128
CD38 PerCP-eF710	Invitrogen	46-0388-42	HB7
CD19 APC-Fire750	BioLegend	302258	HIB19
CD86 Pacific Blue	BioLegend	305418	IT2.2
CD71 BV510	BD OptiBuild	744926	L01.1
CD3 BV570	BioLegend	300436	UCHT1
CD21 PE-Dazzle594	BioLegend	354921	Bu32
CD83 PE	BioLegend	305322	HB15e
CD69 APC	BD Biosciences	340560	L78

Suppl. Table 2. List of differentially expressed genes in peripheral blood purified CD19+ B cells of HBV and HCV patients as compared to CTRL. Genes that are highly expressed by B cells in the CTRL group are shown in positive fold changes. Only significant results are shown (p.adj<0.05).

Suppl. Table 3. Patient characteristics of flow cytometric data from peripheral blood B cells.

	Unit	CTRL	HBV	HCV
Samples	N	9	10	9
Age	Median, IQR	35 (8)	35 (13)	44 (9)
Male	N, %	6 (66.7%)	5 (50%)	5 (55.5%)
Ethnicity	N, %			
Caucasian		4 (44.4%)	1 (10%)	3 (33.3%)
Asian		3 (33.3%)	7 (70%)	4 (44.4%)
African		2 (22.2%)	0 (0%)	1 (11.1%)
Other		0 (0%)	2 (20%)	1 (11.1%)
ALT (U/L)	Median, IQR	NA	43 (54)	45 (27)
Viremia (IU/ml)	Log10(median), IQR	NA	8.5 (1.5)	6.3 (0.4)
HBeAg positive	N, %	NA	10 (100%)	NA
Anti-HBe positive	N, %	NA	0 (0%)	NA
HBV genotype	N,%	NA		NA
	A		2 (20%)	
	B		1 (10%)	
	C		5 (50%)	
	D		1 (10%)	
	ND		1 (10%)	
HCV genotype	N, %	NA	NA	
	HCV-1			1 (11.1%)
	HCV-1a			2 (22.2%)
	HCV-1b			5 (55.5%)
	HCV-4			1 (11.1%)
Fibrosis	N, %			
F0/F1		-	7 (70%)	6 (66.7%)
F2		-	3 (30%)	3 (33.3%)

Suppl. Table 4. Overview of the results obtained by flow cytometric analysis of B cell subsets in blood of chronic HBV, chronic HCV, and CTRL. Results are summarized as median % of parent (IQR). P values were calculated with the Wilcoxon rank-sum test.

**Population**	**CTRL**	**HBV**	**HCV**	**P value**
Lymphocytes/B cells	18.6 (9.1)	18.9 (9.3)	17.5 (10.4)	0.616
B cells/Naive	71.5 (3.5)	77.8 (22.4)	61.9 (39.3)	0.721
B cells/RM	24.8 (4.7)	16.9 (15.9)	30.4 (38.6)	0.429
B cells/AM	1.4 (1.0)	2.1 (1.9)	1.6 (3.0)	0.446
B cells/AtM	1.9 (1.0)	4.8 (2.9)	3.9 (3.2)	**0.0437***
**CD38+**				
Naive	78.2 (12.7)	81.5 (7.0)	78.7 (15.1)	0.5
RM	36.0 (10.0)	37.6 (12.9)	46.9 (19.1)	0.538
AM	9.7 (8.4)	15.9 (6.8)	15.6 (7.1)	0.248
AtM	15.3 (11.9)	23.9 (12.9)	18.8 (10.2)	0.745
**CD38-high**				
Naive	2.4 (2.4)	3.4 (5.2)	2.0 (3.8)	0.908
RM	0.2 (0.5)	0.3 (0.5)	0.2 (0.3)	0.287
AM	7.9 (15.0)	7.2 (6.9)	4.6 (2.7)	0.065
AtM	5.9 (5.6)	6.3 (7.1)	6.0 (7.8)	0.908
**CD69+**				
Naive	3.6 (2.2)	1.7 (1.2)	3.9 (2.3)	0.093
RM	4.2 (2.5)	2.2 (0.8)	3.8 (3.1)	0.279
AM	7.2 (6.5)	5.0 (1.8)	6.8 (3.6)	0.523
AtM	7.5 (3.6)	6.8 (9.1)	9.9 (7.2)	0.628
**CD71+**				
Naive	0.2 (0.4)	0.2 (0.2)	0.3 (0.2)	0.895
RM	1.1 (2.3)	1.7 (1.0)	1.2 (1.7)	0.822
AM	10.8 (8.4)	8.4 (3.9)	11.3 (6.3)	0.609
AtM	3.4 (5.3)	2.5 (1.7)	2.9 (3.7)	0.696
**CD83+**				
Naive	0.3 (0.1)	0.3 (0.2)	0.4 (0.1)	0.277
RM	0.3 (0.1)	0.2 (0.1)	0.2 (0.1)	0.251
AM	0.3 (0.1)	0.2 (0.1)	0.2 (0.4)	0.164
AtM	0.3 (0.2)	0.2 (0.1)	0.2 (0.2)	0.49
**CD86+**				
Naive	0.2 (0.1)	0.2 (0.1)	0.2 (0.1)	0.808
RM	0.3 (0.3)	0.4 (0.2)	0.3 (0.4)	0.981
AM	3.9 (4.8)	3.4 (1.4)	2.3 (4.2)	0.382
AtM	3.4 (5.5)	1.7 (0.8)	1.8 (0.9)	0.101

RM: resting memory, AM: activating memory, AtM: atypical memory. ***Significant in CTRL vs. HBV.*

Suppl. Table 5. Patient characteristics of RNA-seq data from liver biopsies.

	**Unit**	**CTRL**	**HBV**
Samples	N	9	15
Age	Median, IQR	52 (29)	24 (9)
Male	N, %	4 (44.4%)	5 (33%)
Ethnicity	N, %		
Caucasian		7 (77.8%)	2 (13.3%)
Asian		0 (0%)	11 (73.3%)
African		0 (0%)	2 (13.3%)
Other		2 (22.2%)	0 (0%)
ALT (U/L)	Median, IQR	24 (23)	68 (118)
Viremia (IU/ml)	Log10(median), IQR	-	8.6 (1.3)
HBeAg positive	N, %	-	15 (100%)
Anti-HBe positive	N, %	-	0 (0%)
HBV genotype	N,%		
	A	-	1 (6%)
	B	-	3 (18%)
	C	-	7 (46%)
	D	-	3 (18%)
	E	-	1 (6%)
Fibrosis	N, %		
F0/F1		9 (100%)	9 (60%)
F2		0 (0%)	6 (40%)

Suppl. Table 6. List of differentially expressed genes in RNA-seq data of liver biopsies comparing HBV vs. CTRL. Genes that are highly expressed in HBV as compared to CTRL are shown in positive fold changes. Only significant results with an absolute fold change of 1.5 or higher are shown (p.adj<0.05).

Suppl. Table 7. B cell-related genes that are differentially expressed in liver biopsies of HBV as compared to CTRL. Genes that are highly expressed in HBV as compared to CTRL are shown in positive fold-changed. Only significant results with an absolute fold change of 1.5 or higher are shown (p.adj<0.05).

Gene	baseMean	log2FC	lfcSE	stat	pvalue	padj
IGLV1-40	166.9	4.59	0.95	4.85	1.22E-06	5.84E-04
IGHV3-20	34.3	4.37	1.36	3.21	1.33E-03	1.98E-02
IGKV1-17	101.1	3.89	0.99	3.92	8.93E-05	4.74E-03
IGHV1-46	49.5	3.81	1.07	3.56	3.72E-04	9.67E-03
IGHV3-73	36.2	3.73	1.24	3.01	2.60E-03	2.92E-02
IGHV5-51	129.7	3.51	0.87	4.06	5.00E-05	3.38E-03
IGHV3-74	85.9	3.44	0.90	3.82	1.35E-04	5.84E-03
IGHG4	1728.5	3.43	0.60	5.73	1.00E-08	2.15E-05
IGKV1-6	232.8	3.32	1.05	3.17	1.51E-03	2.13E-02
IGHJ4	37.2	3.23	1.10	2.95	3.23E-03	3.32E-02
IGHV4-59	348.9	3.20	0.90	3.55	3.80E-04	9.79E-03
IGHJ3P	157.1	3.16	0.66	4.82	1.43E-06	6.49E-04
IGHV3-43	41.7	3.14	1.13	2.78	5.49E-03	4.59E-02
IGHV3-33	117.8	3.08	1.00	3.07	2.17E-03	2.64E-02
IGLV2-11	115.5	3.05	0.94	3.26	1.12E-03	1.81E-02
IGKV1-12	88.2	2.95	0.98	3.03	2.46E-03	2.82E-02
IGHV3-48	131.5	2.94	0.98	3.01	2.65E-03	2.95E-02
CD79B	84.4	2.89	0.76	3.78	1.54E-04	6.22E-03
IGHJ5	59.8	2.69	0.90	2.99	2.81E-03	3.05E-02
IGLV3-19	119.1	2.67	0.97	2.74	6.16E-03	4.90E-02
IGHA2	283.1	2.61	0.59	4.44	8.79E-06	1.39E-03
IGKV3-20	387.4	2.52	0.92	2.73	6.25E-03	4.94E-02
IGHGP	238.0	2.49	0.73	3.42	6.18E-04	1.30E-02
IGHV4-34	69.8	2.42	0.86	2.80	5.05E-03	4.34E-02
IGHG2	1461.2	2.42	0.52	4.65	3.38E-06	9.81E-04
IGHM	1206.6	2.37	0.36	6.52	7.21E-11	1.11E-06
IGKV1-5	248.6	2.35	0.75	3.14	1.70E-03	2.29E-02
IGHV3-23	229.0	2.34	0.76	3.10	1.94E-03	2.47E-02
IGHV4-61	82.8	2.31	0.83	2.77	5.58E-03	4.61E-02
IGKV4-1	194.8	2.23	0.75	2.96	3.04E-03	3.21E-02
IGHA1	1051.2	1.73	0.44	3.97	7.20E-05	4.20E-03
SLAMF7	677.4	1.52	0.36	4.22	2.41E-05	2.26E-03
CD38	1056.0	1.33	0.34	3.96	7.49E-05	4.32E-03

Suppl. Table 8. List of differentially expressed genes in single-cell RNA-seq data of B cells from liver FNAs of immune active HBV vs. CTRL. Genes that are highly expressed by intrahepatic B cell clusters in the CTRL group are shown in positive fold changes. Results per cluster can be found within separate sheets. Only significant results with an absolute fold change of 1.5 or higher are shown (p.adj<0.05).
